# Early Stepdown Weaning of Dairy Calves with Glutamine and Branched-Chain Amino Acid Supplementations

**DOI:** 10.3390/ani12121474

**Published:** 2022-06-07

**Authors:** Janaka Wickramasinghe, Can Ayhan Kaya, Donald Beitz, Ranga Appuhamy

**Affiliations:** 1 Department of Animal Science, Iowa State University, Ames, IA 50011, USA; janaka@iastate.edu (J.W.); dcbeitz@iastate.edu (D.B.); 2 Department of Livestock and Crop Production, Dicle University, Diyarbakir 21280, Turkey; canayhan.kaya@dicle.edu.tr

**Keywords:** branched-chain amino acids, dairy calves, glutamine, starter feed intake

## Abstract

**Simple Summary:**

We demonstrated previously that supplementation of glutamine (Gln) at 2.0% of dry matter intake (DMI) increased the rate at which dairy calves achieved ≥1.0 kg/d starter feed intake (SFI) during weaning. Because Gln supplements at <1.0% of DMI or branched-chain amino acid (BCAA) supplements have been shown to improve the performance of weaning piglets, we examined the effects of a lower dose of Gln (8.0 g/d equivalent to 1% of DMI) alone or in combination with BCAA supplementations on SFI and average daily gain (ADG) in this study. Amino acids did not affect SFI or ADG during the supplementations but decreased post-weaning SFI in an additive manner even though the ADG was not affected. The blood analysis on the last day of supplementations revealed a possibility for the Gln and BCAA supplementations to suppress SFI through leptin and serotonin secreted by the gastrointestinal tract.

**Abstract:**

The study objective was to examine the effects of supplementing Gln and BCAA on the SFI and ADG of weaning dairy calves. Holstein heifer calves (11 calves /treatment) at 35 d of age were assigned to: (1) no amino acids (CTL), (2) Gln (8.0 g/d) alone (GLN), or (3) Gln (8.0 g/d) and BCAA (GLNB; 17.0, 10.0, and 11.0 g/d leucine, isoleucine, and valine, respectively) supplementations in whole milk during a stepdown weaning scheme. Calves were weaned completely once they achieved ≥1.0 kg/d SFI. Neither GLN nor GLNB affected SFI or ADG in the first week during weaning. The GLNB decreased SFI compared to CTL, but the SFI was similar between CTL and GLN in the remainder of the weaning scheme. All calves were weaned at 50 d of age. The SFI of GLNB was lower than that of GLN, and the SFI of both GLN and GLNB were lower than CTL post-weaning. The decreased SFI did not alter ADG during weaning or post-weaning. The GLNB tended to have higher plasma leptin and lower plasma serotonin concentrations compared to CTL. Glutamine and BCAA seem to affect the SFI of calves by modulating the secretions of endocrine cells in the gastrointestinal tract.

## 1. Introduction

Recent surveys indicate that most dairy producers in the U.S. wean calves at 9 weeks of age [1,2]. Weaning at an earlier age is profitable as the cost of raising pre-weaned calves is notably higher than that of weaned calves (USD 6.50 vs. USD 3.00/calf daily, [3,4]). Early weaning schemes should not, however, entail severe decreases in nutrient intake as this could exacerbate weaning stresses [5,6]. Weaning from a high milk volume (>8.0 kg/d) is particularly challenging as calves experience a prolonged lag in achieving the desired SFI post-weaning [7,8,9]. Hence, stepdown weaning schemes are proposed to encourage satisfactory SFI at weaning (0.9 to 1.4 kg/d) [10,11]. Eckert et al. [12] employed a stepdown weaning scheme where the milk replacer allowance was restricted (8.0 to 4.0 L/d) at 35 d, and calves were weaned completely at 42 d, regardless of SFI. In that scheme, calves had lower ADG during weaning as well as post-weaning compared to calves that were weaned 14 d later. Wickramasinghe et al. [13] employed a similar scheme but avoided the post-weaning growth slump by delaying complete weaning until calves achieved a 1.0 kg/d SFI. Moreover, Wickramasinghe et al. [13] demonstrated that supplementing Gln (2.0% of DMI) increased ADG during weaning and helped calves achieve the 1.0 kg/d SFI earlier than calves that were not supplemented with Gln. 

Despite the general acceptance as a non-essential amino acid (NEAA), Gln can be conditionally essential as endogenous Gln production becomes inadequate in times of stress and illness [14]. Glutamine is a predominant energy substrate of enterocytes, and Gln deficiency exacerbates the weaning-induced damage to the intestinal barrier function and absorptive capacity of the small intestine [14,15]. Wickramasinghe et al. [13] showed that supplementation of Gln alleviated the damage to the paracellular permeability of intestinal epithelium of calves undergoing weaning at 35 d of age. The authors speculated that offering some volume of whole milk until calves consumed ≥1.0 kg/d SFI led to a successful recovery from early weaning without negatively affecting SFI and ADG post-weaning. This speculation is reasonable because milk is rich in various bioactive compounds and functional amino acids such as Gln and BCAA that could mitigate the adverse effects of weaning stresses [16,17]. 

The BCAA, including isoleucine (Ile), leucine (Leu), and valine (Val) are essential amino acids (EAA) that must be obtained through diet, as the body cannot synthesize them in adequate quantities. Besides being the building blocks for protein synthesis, BCAA serve as cellular signals. They stimulate protein synthesis and cell proliferation and play critical roles in the growth and development of young calves [17,18]. The BCAA could limit growth during weaning as serum BCAA concentrations decreased more rapidly in calves with positive ADG during weaning than calves with no ADG during weaning [13]. Ren et al. [19] demonstrated that supplementing a low-protein diet with BCAA increased feed intake and the ADG of weaning piglets, which could be positively correlated with the future growth performances [20]. Overall, the supplementation of each Gln and BCAA seems to help young animals cope with weaning stresses as reflected in enhanced growth and feed intake at weaning, which can have a positive impact on post-weaning performances. In the present study, we hypothesized that the supplementation of BCAA with Gln would enhance the SFI and ADG of weaning calves compared to the supplementation of Gln alone. The study objective was to examine the effects of supplementing Gln alone or in combination with BCAA on SFI, ADG, and the blood metabolites of dairy heifer calves undergoing stepdown weaning from a high milk volume at an early age. The roles of dietary protein and amino acids in calves are poorly understood. Because Gln and BCAA are the most abundant amino acids in milk protein, this study could improve the understanding of the roles of milk protein in regulating SFI and the growth of dairy calves.

## 2. Materials and Methods

### 2.1. Animals and Treatments

All experimental procedures were approved by the Animal Care and Use Committee at Iowa State University, Ames, IA, USA (IACUC# 19–172). The experiment was conducted at the Dairy Research and Teaching Farm at Iowa State University (Ames, IA, USA). Thirty-three Holstein heifer calves that were born and raised in the Iowa State University Dairy Teaching and Research Farm were enrolled in the experiment. All calves were bottle-fed at least 3.80 kg of thawed colostrum within the first 4 h of life. Colostrum was collected from cows at the farm, pooled, aliquoted, and frozen in plastic bags (Dairy Tech Inc., Greeley, CO, USA). Calves were bottle-fed with 6.0 kg/d of pasteurized whole milk (2.0 kg at each of 0600, 1400, and 2200 h feeding) until 14 d of age. From d 14 onward, the milk volume was increased to 9.0 kg/d (3.0 kg per feeding) until 35 d of age. After blocking for dam’s parity and birth-week, the 33 calves (28 d of age) were assigned to 3 treatments (11 calves per treatment; *n* = 11), involving a stepdown weaning scheme, where the milk allowance was restricted to 3.0 kg/d at 35 d of age, and calves were weaned completely once they achieved ≥1.0 kg/d SFI over two consecutive days. The treatments were: (1) no amino acid supplementation (CTL), (2) supplementation of Gln alone (GLN), and (3) supplementation of Gln and BCAA (GLNB) in milk that was offered to individual calves. In this paper, the terms “pre-weaning”, “during weaning”, and “post-weaning” describe the week immediately before initiating weaning on 35 d, the period from 36 d to the completion of weaning, and the period after the completion of weaning, respectively. Amino acid (AA) supplements were fed in milk over the last 3 d pre-weaning and in the first week during weaning (33 to 42 d of age). The Gln supplementation was 8.0 g/d, which was predicted to be equivalent to 1.0% of the DMI (milk solid and starter dry matter) of calves [13]. The 1.0% supplementation was chosen as Gln has been shown to improve the weaning performances of piglets with supplementations ≤1.0% of dry feed intake. The BCAA supplements (L-leucine (17.0 g/d), L-isoleucine (10.0 g/d), and L-valine (11.0 g/d)) were determined to be equivalent to the amounts and the ratio of Leu, Ile, and Val in milk protein [21] in the 6.0 kg of cow milk that was removed from the diet with the initiation of weaning. Therefore, the prospective effects can be related to BCAA in milk protein (e.g., leucine: isoleucine: valine). The free AA in powdered form were dissolved in milk (3.0 kg) by shaking the bottles thoroughly for about 10 s before offering to the calves at 2200 h feeding. All calves were housed in individual pens bedded with straw (floor area = 1.2 m × 1.8 m). All calves had access to ad libitum amounts of drinking water and a texturized calf starter with whole corn grain (Vita Plus Corp., Madison, WI, USA) throughout the study. An ad libitum starter intake was maintained by offering 10% more starter that was relative to the intake from the previous day. Starter feed samples collected weekly were composited within every month, and the composited samples were analyzed for dry matter (DM), ether extract, acid detergent fiber (ADF), crude protein (CP) [22], and neutral detergent fiber (NDF) [23] at Cumberland Valley Analytical Services (Waynesboro, PA, USA). According to analysis results, the calf starter contained 89.2% DM and 24.0, 3.9, 20.2, and 12.8% of CP, crude fat, NDF, and ADF, respectively.

### 2.2. Measurements and Sample Collection

The body weight (BW), hip height, body length, and hip width of individual calves were measured weekly by the same person immediately before evening (2200 h) feeding. The weights of the milk, calf starter, and drinking water that were offered and leftover (kg) were recorded daily. The blood (8.0 mL/tube) was collected with jugular venipuncture (18-gauge 1 inch (2.54 cm) long needles) into two vacutainer tubes (Becton, Dickinson and Company, Franklin Lakes, NJ, USA); one tube contained K_2_EDTA and the other had no anticoagulant, and the samples were taken on 35 d and 42 d before evening feeding (2200 h). The serum and plasma were separated by centrifuging at 2000× *g* at 4 °C for 20 min and stored at −20 °C until analyses were performed. 

#### 2.2.1. Haptoglobin Analysis 

Serum haptoglobin (HPT) concentrations on 35 and 42 d were measured using a commercial kit (Life Diagnostics Inc., West Chester, PA, USA, catalog# HAPT-11) following the manufacturer’s protocol. Briefly, serum samples were diluted (1:50) first with the diluent of the kit. The diluted samples and the standards (100 µL) were transferred in duplicate to a 96-well plate and then incubated on an orbital shaker (150 rpm) at room temperature for 45 min. Then, 100 µL of a horseradish peroxidase conjugate was added to each well and incubated on the orbital shaker (150 rpm) at room temperature for 45 min. Tetramethylbenzidine substrate (100 µL) was dispensed to each well, and the plate was incubated on the orbital shaker (150 rpm) at room temperature for 20 min. After adding the stop solution (100 µL) to each well, the optical density was read at 450 nm by using an Eon microplate spectrophotometer (BioTek Instruments, Inc., Winooski, VT, USA). The samples were reanalyzed if the coefficient of variation (CV) across duplicates was >20%. The intra-assay CV was <10% for 90% of the samples, and the inter-assay CV was 2.0%. The detection limit of this assay was 4.0 ng/mL.

#### 2.2.2. Leptin Analysis 

The plasma leptin (LPN) concentration was measured on 42 d with an ELISA kit of mouse leptin (~90% homology with bovine leptin, [24]) following the manufacturer’s (MilliporeSigma, Burlington, MA, USA, catalog# RAB0334) protocol. Briefly, the plasma samples were diluted (1:5) and transferred (100 µL) in duplicate to a 96-well plate that was incubated on an orbital shaker (150 rpm) at 4 °C overnight. The detection antibody (100 µL) was added to each well and incubated again on the shaker (150 rpm) at room temperature for 1 h. The streptavidin solution (100 µL) was added to each well. After 45 min of incubation (150 rpm) at room temperature, tetramethylbenzidine (100 µL) was dispensed into each well. The plates were incubated on the shaker (150 rpm) at room temperature for 30 min. After adding the stop solution to each well (50 µL), the optical density was read at 450 nm using an Eon microplate spectrophotometer (BioTek Instruments, Inc.). The intra-assay CV of 60% of the samples was <10%, and the rest had a CV of <20%. The detection limit of the assay was 4.0 pg/mL.

#### 2.2.3. Serotonin Analysis 

The plasma serotonin (STN) concentration was measured on 42 d using a commercial kit (Beckman Coulter, Brea, CA, USA, catalog# IM1749) according to the manufacturer’s protocol. Briefly, the plasma samples were diluted (1:200) and transferred into tubes containing acylating reagent. After adding acylation buffer (50 µL), the tubes were incubated at room temperature in the dark for 30 min. The samples (20 µL) were then transferred in duplicate to a 96-well plate. After adding serotonin-acetylcholinesterase conjugate (200 µL) into each well, the plate was incubated at room temperature for 3 h. After adding 200 µL of the substrate into each well the plate was incubated again at room temperature for 20 min. The stop solution (50 µL) was added and the optical density was read at 405 nm using an Eon microplate spectrophotometer (BioTek Instruments, Inc.). The samples were reanalyzed if the CV across duplicates was >20%. The intra-assay CV of 90% of the samples was <10%, and the rest had CV <20%, while the inter-assay CV was 12%. The detection limit of the assay was 0.58 nmol/L.

#### 2.2.4. Analyses of Amino Acids and other Metabolites 

Concentrations of serum metabolites, including AA, glucose, beta-hydroxybutyrate (BHB), and urea on 35 and 42 d were determined with GC-MS at the W. M. Keck Metabolomics Research Laboratory at Iowa State University (Ames, IA, USA). Briefly, ribitol (10 µL from 1 mg/mL stock) was added to 100 µL of each serum sample as an internal standard for polar compounds. On the other hand, 10 µL of nonadecanoic acid (1 mg/mL) was added to the same serum sample as an internal standard for nonpolar compounds. After adding 0.9 mL of methanol (8:2 MeOH:H_2_O), the serum samples for both polar and nonpolar compound analyses were incubated for 10 min on ice, followed by a 10-min sonication in an ice-cold water bath. The samples were centrifuged for 7 min at 4 °C and 13,000× *g*, and 500 µL of the supernatant was transferred into GC-MS vials and dried in a SpeedVac concentrator (Savant SpeedVac SPD120 Vacuum Concentrator, Thermo Fisher Scientific, Waltham, MA, USA) overnight. Next, 50 µL of methoxyamine hydrochloride (20 mg/mL) were added, and samples were incubated at 30 °C for 1.5 h. Trimethylsilylation was performed by incubating samples with 70 µL of bis-trimethyl silyl trifluoroacetamide containing 1% trimethylchlorosilane for 30 min at 60 °C. The GC-MS analyses were performed on 1.0 µL of the sample by using an Agilent 6890N GC that was coupled to a mass selective detector (model 5975, Agilent Technologies, Santa Clara, CA, USA). The column used was 5% phenyl methyl siloxane with a 30 m × 250 μm × 0.25 μm film thickness (Agilent 19091S-433, Agilent Technologies). The oven temperature was increased first from 50 to 245 °C at a rate of 5 °C/min and then to 320 °C at a rate of 20 °C/ min. Helium was used as a carrier gas at a 1.2 mL/min initial flow rate. The quantifications were performed by using electron ionization at 70 eV by setting the source and quadrupole temperatures at 230 °C and 150 °C, respectively. The mass data were collected from m/z 50 to m/z 800. An Agilent Enhanced ChemStation version D.02.00.275 was used for the identification of compounds. Peak detection and deconvolution were performed using AMDIS software. The peaks were identified by comparing them to the spectra and retention index data in NIST17/Wiley11 libraries. The abundance of compounds was quantified by integrating the corresponding peak areas that were relative to the area of the internal standards.

### 2.3. Calculations and Statistical Analysis

The sample size was determined with a power analysis (power = 0.80 and α = 0.05) to capture the increase in the average SFI of calves undergoing the present weaning scheme from 0.55 [13] to 1.0 kg/d [10,11] given that the standard deviation of SFI was 0.36 kg/d [13]. Treatment effects on the responses of interest were determined by using the following statistical model:

Y_ijklm_ = μ + T_i_ + P_j_ + R_k_ + W_l_ + (T × P)_ij_ + C_m_+ e_ijklm_,

where Y_ijklm_ = the response variable of interest; μ = the overall mean of the response; T_i_ = the fixed effect of the ith treatment (i = CTL, GLN, and GLNB); P_j_ = the fixed effect of the jth period (j = pre-weaning, during weaning, and post-weaning); R_k_ = the fixed effect of the kth parity of the dam (k = primiparous and multiparous); W_l_ = the fixed effect of the lth week born (l = 1, 2, 3,…, 8); (T × P)_ij_ = the fixed effect of the interaction between the ith treatment and jth period; C_m_ = the random effect of the mth calf; and e_ijklm_ = the random error assumed to be independent and identically distributed from a normal distribution with a mean of 0 and a variance of σ^2^ (~N (0, Iσ^2^)). In the SFI and drinking water intake analyses, the post-weaning period was defined including the daily observations of 14 d immediately following complete weaning. Using this method, every calf had the same number of daily observations for that period. When the SFI and drinking water intake were analyzed, pre-weaning SFI (29 to 32 d) was used as a covariate in the model. When the growth performances were analyzed, the model included the baseline BW (28 d of age) as a covariate. All analyses were performed by using the MIXED procedure of SAS (version 9.4, SAS Institute Inc., Cary, NC, USA). The REPEATED option and the compound symmetry variance–covariance structure were used in all analyses except that of weaning age, ADG: SFI at 70 d, LPN, and STN. The treatment effects on those variables were tested excluding the fixed effect of period and treatment × period interaction from the model as a single calf provided only one observation. The least squares means were compared using the Tukey–Kramer adjustment test. The *p*-value for the overall treatment effect within a given period was obtained by using the SLICE option. Additionally, the orthogonal contrasts of CTL vs. GLN + GLNB and GLN vs. GLNB were performed for the SFI and growth responses. The significant differences and tendencies were declared at *p* ≤ 0.05, and 0.05 < *p* ≤ 0.10, respectively.

## 3. Results

### 3.1. Intake and Growth

Treatment effects on SFI, drinking water intake, and the growth performance in pre-weaning, during weaning, and post-weaning periods are shown in Table 1. The treatment × period interaction affected both starter feed and drinking water intake (*p* < 0.01, data not shown) and ADG (*p* = 0.05). Data did not support such an interaction on BW and body frame measurements (*p* > 0.10, data not shown). The contrasts were not significant (*p* > 0.14), except for the GLN vs. GLNB (*p* = 0.02) of ADG during the first week of weaning, and CTL vs. GLN+GLNB (*p* < 0.01) and GLN vs. GLNB (*p* = 0.03) of SFI post-weaning (data not shown).

#### 3.1.1. Pre-Weaning

**Figure 1:**
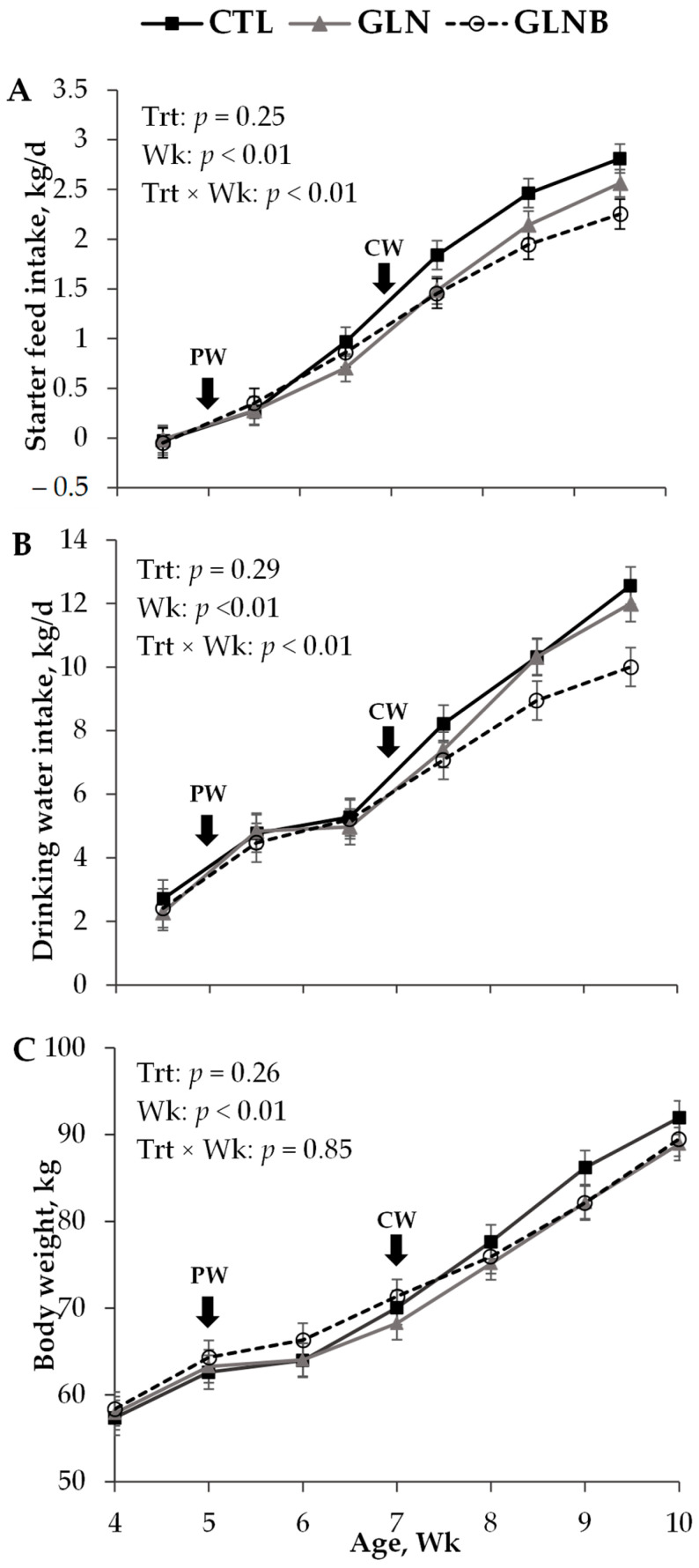
Weekly (Wk) mean (± SEM) starter feed intake (**A**), drinking water intake (**B**) and body weight (**C**) of calves undergoing a stepdown weaning scheme without AA supplementation (CTL), and with Gln alone (GLN) or Gln and branched-chain amino acid (GLNB) supplementations (*n* = 11) as treatments (Trt). PW = the initiation of stepdown weaning by restricting the milk allowance from 9.0 to 3.0 kg/d at 35 d of age, CW = complete weaning as starter intake approached 1.0 kg/d.

Regardless of treatments, the calves consumed a negligible amount of starter feed (0.08 ± 0.08 kg/d, Table 1 and Figure 1A) but drank 2.40 kg/d water from buckets (Table 1 and Figure 1B). All calves had similar BW (63.4 kg, *p* = 0.81, Figure 1C), body frame measurements (*p* > 0.48, Table 1), and ADG (0.85 kg/d, *p* = 0.92, Table 1) pre-weaning. 

#### 3.1.2. During Weaning 

Once weaning was initiated by restricting the milk volume, SFI increased (*p* < 0.01), but calves had a similar SFI across the treatments in the first week during weaning (0.43 kg/d, *p* = 0.42). Drinking water intake also increased with the milk volume restriction (4.82 vs. 2.37 kg/d, *p* < 0.01). The drinking water intake of GLNB was lower than that of CTL in the first week during weaning (4.32 vs. 5.28 kg/d, *p* = 0.05). The CTL and GLN had similar (*p* = 0.48) but negligible ADG, whereas GLNB had a 0.32 kg/d ADG that was greater than that of GLN (GLN vs. GLNB contrast, *p* = 0.02) in the first week during weaning. Once the AA supplementation ceased on 42 d, all calves increased further SFI (*p* < 0.01). The CTL and GLN had a similar SFI (*p* = 0.15), whereas the SFI of GLNB was lower than that of CTL (0.68 vs. 0.89 kg/d, *p* = 0.03) in the remainder of the days during weaning. All calves achieved ≥1.0 kg/d SFI and were thus weaned completely at a similar age of 50 d (*p* = 0.90). Despite the SFI increase, the drinking water intake of the rest of the days remained unchanged from that of the first week during weaning (4.82 vs. 4.54 kg/d, *p* = 0.23). The GLNB drank less water compared to CTL in the remainder of the days during weaning (*p* < 0.01). All calves had a similar ADG in the rest of the days (*p* = 0.27), which was higher than that in the first week during weaning (*p* < 0.01). Moreover, the BW (69.4 kg) and body frame measurements recorded at 49 d, which was close to the weaning age, were similar between treatments (*p* > 0.60, Table 1). 

#### 3.1.3. Post Weaning

The GLNB had lower a SFI compared to CTL (19%, *p* < 0.01) in the first 14 d post-weaning (Table 1). Moreover, the SFI of GLN was lower than that of CTL (9%, *p* < 0.01) and higher than that of GLNB (10%, *p* < 0.01), suggesting the additive but negative effects of GLN and BCAA supplementations during weaning on the SFI post-weaning (Figure 1A). The drinking water intake of CTL and GLN were similar (*p* = 0.99) but greater than that of GLNB (*p* < 0.01) post-weaning. The difference in drinking water intake between CTL and GLNB seemed to increase with time post-weaning (Figure 1B). The BW and body frame measurements were similar between treatments at 70 d of age (*p* > 0.40). The GLNB tended to have a higher ADG (1.08 vs. 0.77, *p* = 0.08) and had a higher ADG: SFI (0.50 vs. 0.29, *p* < 0.01) in the last week (63 to 70 d of age) compared to CTL. 

### 3.2. Metabolites, Haptoglobin, Serotonin, and Leptin Concentrations in the Blood

**Figure 2:**
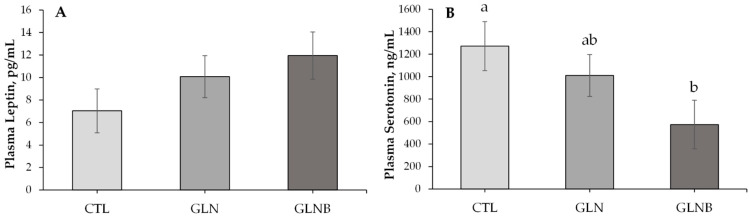
Plasma leptin (**A**) and serotonin (**B**) concentrations of weaning calves (*n* = 11) on the last day (42 d of age) of supplementing Gln (GLN) or Gln with BCAA (GLNB) compared to weaning without an AA supplementation (CTL). ^a,b^ Least squares means with different superscripts indicate significant difference (*p* < 0.05).

The serum AA, glucose, BHB, and urea concentrations that were measured on 1 d before (pre-weaning) and 7 d after the initiation of weaning (during weaning) are presented in Table 2. There were no treatment × time effects on any of those metabolites except Ala (*p* = 0.06), the concentration of which was lower in GLNB compared to GLN during weaning (*p* = 0.04), while GLN and GLNB had similar Ala concentrations pre-weaning (*p* = 0.57). Supplementation of Gln alone or with BCAA did not change serum Gln + Glu concentrations (*p* > 0.94). However, Gln + Glu tended to increase (36%) during weaning compared to pre-weaning concentrations (*p* = 0.08). The serum BHB concentration of GLN decreased compared to that of CTL (*p* < 0.01) but was similar to that of GLNB (*p* = 0.23) during weaning. Nonetheless, the serum BHB concentration was higher (65%) during weaning compared to the pre-weaning concentration (*p* < 0.01). The serum glucose concentration decreased (6.35 to 5.57 mmol/L, *p* < 0.01), whereas the serum urea concentration increased (0.59 to 0.68 mmol/L, *p* = 0.02) during weaning compared to pre-weaning concentrations. The GLN or GLNB did not affect serum glucose or urea concentrations during weaning or pre-weaning (*p* > 0.20, Table 2). Serum HPT concentrations were not different between treatments (*p* = 0.52, Table 2) but tended to increase (0.65 to 0.86 µg/mL, *p* = 0.07) during weaning as opposed to the pre-weaning concentration. Plasma LPN and STN concentrations that were measured at the end of AA supplementation are presented in Figure 2A,B, respectively. The plasma LPN concentrations were similar between CTL and GLN (*p* = 0.58, Figure 2A). Plasma LPN tended to increase over CTL (*p* = 0.07, Figure 2A) in response to GLNB (Figure 2A). The GLN did not affect the plasma STN concentration relative to CTL (*p* = 0.87, Figure 2B), whereas GLNB decreased the STN relative to CTL (*p* = 0.04) and tended to decrease it relative to GLN (*p* = 0.06, Figure 2B). 

## 4. Discussion

Glutamine and BCAA are involved in physiological mechanisms regulating food intake and growth and are referred to as functional amino acids [16]. Although BCAA and Gln are abundant in milk protein [25], their impact on the feed intake and growth of calves is poorly understood. Wickramasinghe et al. [13] demonstrated that supplementing Gln (2.0% of DM) helped calves to achieve ≥1.0 kg/d SFI earlier than calves without Gln even though the ADG was unaffected during weaning. The data on BCAA supplementations for weaning calves are hard to find, but BCAA supplementations have improved the ADG of weaning piglets [19]. Therefore, in the present study, the BCAA representatives of leucine: isoleucine: valine of milk protein were supplemented in addition to Gln to enhance the SFI and ADG of weaning calves. The calves underwent stepdown weaning from a high milk volume, and about 30% of pre-weaning dairy calves in the U.S. consume high milk or milk replacer volumes [1]. However, the Gln dose was decreased (2.0 to 1.0% of DMI) from the previous study [13] as Gln supplementations at ≤1.0% of DMI have been shown to improve the ADG and feed intake of weaning piglets [26] and weaning calves [27].

The present study results attest that calves who were offered high milk volumes consumed a negligible amount of starter feed pre-weaning. On the other hand, they consumed 2.4 kg/d of free water, highlighting the importance of unlimited access to drinking water despite being fed high milk volumes [28]. Similar to what has been observed previously [13], the milk restriction halted growth in the first week during weaning. Other than the nutrient restriction, nutrient repartitioning to support other functions than growth could partly account for the growth impairment at the onset of weaning. In support, the increased serum HPT suggests an increased energy partitioning towards an immune activation [29,30,31]. Nevertheless, similar serum HPT concentrations among treatments do not corroborate an involvement of Gln or BCAA in modulating such immune activation during weaning. Serum Gln + Glu concentrations not responding to GLN or GLNB were consistent with Wang et al. [27] who reported unchanged serum Gln and Glu concentrations of weaning dairy calves in response to oral Gln supplementations up to even 4.0% of DMI. Similar to the present study, Wang et al. [27] measured the concentrations about 24 h after feeding AA. Li et al. [32] demonstrated that serum BCAA of Holstein calves (28 d of age) exhibited the postprandial peak 6 h after feeding BCAA, and those concentrations returned to baseline concentrations that were similar to the concentration in control calves 18 h after feeding. The decreased serum BHB of GLN vs. CTL could be more of a result of a decline in fatty acid mobilization from adipose tissues [33,34] than a volatile fatty acid production decline in the rumen because the SFI of GLN and CTL were similar in the first week during weaning. Drinking water intake: SFI in the first week during weaning was nearly two times higher than that in the remainder of days during weaning (11:1 vs. 6:1). The higher water intake relative to SFI could be a result of the SFI surge that could increase ruminal and blood osmolality at the onset of weaning [35,36]. On the other hand, the lower (18%) drinking water intake of GLNB vs. CTL, despite similar SFI, postulates the involvement of other mechanisms. Perhaps the increased drinking water intake was also a behavioral response to weaning stresses [37], and GLNB lowered those stresses compared to CTL. 

Contrary to our previous study [13] where Gln-supplemented calves achieved ≥1.0 kg/d SFI three days earlier, GLN did not affect the SFI during weaning or weaning age in the present study. Lowering the Gln dose from 2.0 to 1.0% of DMI (14.0 to 8.0 g/d) might partly explain this discrepancy. However, Wang et al. [27] reported the increased SFI of weaning calves fed Gln at 1.0% of DMI, which was equivalent to 14.0 to 18.0 g/d. It is not clear whether the efficacy is related to the actual amount (g/d) or the rate at which Gln is fed (per DMI or BW). Moreover, Wang et al. [27] supplemented Gln to starter feed, whereas we supplemented it to pasteurized whole milk that contained free Gln (not determined) and highly digestible proteins that yield more Gln than the proteins in starter feed. The CTL being yet to be able to provide a considerable amount of Gln might have hampered the likelihood of seeing an impact with the 8.0 g/d or 1.0% of DMI dose of Gln in the present study. Furthermore, the calves in Wang et al. [27] were weaned abruptly from an 8.0 L/d of milk replacer (12.5%, w/v) as opposed to stepdown weaning in the present study. Some literature also suggests that Gln supplementations could be more effective in immune-challenged animals than in healthy animals [38]. Perhaps the present calves had a better health status and underwent a lower degree of stress at weaning than the calves in Wang et al. [27] and the calves in our previous study [13]. In support, the present calves had a higher pre-weaning ADG than that of the previous calves (0.86 vs. 0.46 kg/d). Similar factors could also influence the efficacy of BCAA supplementations in calves. For instance, BCAA infusions improved the feed intake and nitrogen retention of beef steers that were challenged with bacterial lipopolysaccharides (LPS) in Löest et al. [39]. The BCAA infusions had no impact on the performances of steers without LPS in that study. 

The literature does not support the negative and seemingly additive effects of Gln and BCAA supplementations during weaning on the SFI post-weaning. Contrary to the present results, Gln supplements ≤1.0% of DMI during weaning improved the post-weaning feed intake of piglets in Duttlinger et al. [26]. Moreover, Gln supplementation during weaning did not decrease SFI in the previous study [13]. One might speculate that essential nutrients such as BCAA could negate the appetite stimuli if the supplementation exceeds the requirement. The present weaning scheme may represent such a scenario, as the ADG depression likely lowered the requirement. However, the paradox is that GLN and GLNB decreased SFI, not during the AA supplementation, but post-supplementation, suggesting a sustained impact on feed intake regulation. The involvement of AA in feed intake regulation has been described previously [40]. Tian et al. [41] observed that BCAA supplementations to a low-protein diet increased or decreased feed intake, and those effects were dose-dependent. They reported that BCAA regulated feed intake through intestinal and hypothalamic AA receptors. Amino acid sensing by these receptors can stimulate the intestinal endocrine cells to release a variety of hormones that are secreted by endocrine cells in the gastrointestinal tract, such as STN and LPN [42,43,44]. The literature suggests further that STN and LPN can integrate short-term nutritional signals into a long-term regulation of food intake [45]. Although other tissues also produce those hormones, gastrointestinal secretions seem to contribute more to plasma STN and LPN in calves than in mature cattle [46]. Therefore, STN and LPN concentrations in blood that was drawn on the last day of AA supplementations were determined to explain those paradoxical results of SFI. 

In this study, plasma LPN and STN tended to increase and decreased in response to GLNB, respectively, and had a negative relationship with each other (r = −0.37, *p* = 0.05, data not shown). The present plasma STN concentrations (600 to 1200 ng/mL) were lower than previously reported concentrations in the serum or whole blood (2000 to 4000 ng/mL) of calves [47,48]. This is not surprising as the majority of circulating STN is stored in the platelets [49]. The origin of plasma STN is the enterochromaffin cells of the gastrointestinal tract mucosa that produce STN by using tryptophan. Serotonin in the blood, known as peripheral STN accounts for over 95% of the total STN in the body. The balance is produced in the brain and is known as central STN [50,51,52]. The central STN is known well for its appetite suppression effect, whereas some data suggest an appetite-enhancing role for plasma STN. For instance, Goodarzi et al. [53] demonstrated a feed intake drop when plasma STN receptors were blocked in pigs. Moreover, the plasma STN and feed intake of pigs decreased in response to Leu supplementation, while central STN concentration remained unchanged compared to the control in Wessels et al. [54]. Those authors speculated that Leu likely decreased plasma STN by impairing the tryptophan uptake to enterochromaffin cells. The present results support a notion that such mechanisms may have a sustained impact on STN synthesis and thus feed intake. Contrary to peripheral STN, LPN can cross the blood–brain barrier and reach the hypothalamus, where it exerts its appetite-suppressing effects [55,56]. The data supporting the associations of Gln or BCAA supplementations with plasma LPN are sparse. Noaman [57] demonstrated, however, that intravenous STN injection decreased plasma LPN in rats corroborating the negative relationship between plasma STN and LPN. A comprehensive analysis with more longitudinal samples to determine both the hypothalamic and gastrointestinal factors involving feed intake control would provide better insight into how the AA supplementations elicited a sustained and negative impact on the SFI of calves. Such an analysis can also assist in understanding the role of free or protein-bound AA in milk or milk replacer in the SFI control of suckling calves. Future experiments accounting for Gln + Glu in milk, and the immune status and stress intensity of animals would provide better insight into the dose-effect of exogenous Gln in weaning calves. Furthermore, experiments designed to test the effects of not only total but also individual BCAA supplementations can provide a refined understanding of BCAA’s role in the feed intake control of calves. 

## 5. Conclusions

In the present weaning scheme beginning at 35 d of age, Holstein dairy heifer calves fed a high milk volume (9.0 kg/d) were weaned with ≥1.0 kg/d SFI and about 70 kg BW at 50 d of age. The milk restriction (from 9.0 to 3.0 kg/d) negated the ADG in the first week during weaning (35 to 42 d of age). Glutamine supplementation at 8.0 g/d (1.0% of DMI) in the first week during weaning (35 to 42 d) did not affect SFI or ADG compared to CTL during weaning. Adding BCAA (38.0 g/d) with leucine: isoleucine: valine similar to that found in milk protein to the Gln supplementation did not affect SFI but tended to increase ADG (0.09 to 0.31 kg/d) compared to GLN in the first week during weaning. The supplementation of Gln and BCAA decreased the SFI after the supplementation was ceased during weaning, and the SFI declines prevailed post-weaning even though ADG was not affected. The Gln and BCAA supplementations tended to increase plasma LPN and decreased plasma STN concentrations that were measured on the last day of AA supplementations. Further investigations on the involvement of AA in feed intake control would expand the current understanding of the relationships between the nutrient composition of milk or milk replacers and the SFI of dairy calves.

## Figures and Tables

**Table 1:** Performance of dairy heifer calves undergoing a stepdown weaning scheme with AA supplementations (*n* = 11).

Variable	Treatment ^1^			SEM	*p*-Value
	CTL	GLN	GLNB		
Baseline BW (28 d), kg	57.3	57.8	57.7	0.4	0.32
**Pre-weaning (33 to 35 d)**					
Starter feed intake, kg/d	0.08	0.06	0.14	0.08	0.74
Drinking water intake, kg/d	2.62	2.02	2.47	0.42	0.59
Body measurements (35 d) ^2^					
BW, kg	62.6	63.2	64.4	1.9	0.81
HH, cm	86.3	86.5	87.4	0.7	0.48
HW, cm	22.3	22.1	22.6	0.3	0.54
BL, cm	80.1	79.8	81.0	1.1	0.73
ADG, kg/d	0.86	0.83	0.88	0.10	0.92
**During weaning (36 d to complete weaning)**					
***The first week (36 to 42 d of age)***					
Starter feed intake, kg/d	0.46	0.38	0.45	0.04	0.42
Drinking water intake, kg/d	5.28 ^a^	4.85 ^ab^	4.32 ^b^	0.19	<0.01
BW (42 d), kg	64.0	64.0	66.4	1.9	0.62
ADG, kg/d	0.18	0.09	0.32	0.1	0.26
***The remainder of the days***					
Starter feed intake, kg/d	0.89 ^a^	0.73 ^ab^	0.68 ^b^	0.04	<0.01
Drinking water intake, kg/d	4.99 ^a^	4.57 ^ab^	4.13 ^b^	0.19	0.02
Body measurements (49 d)					
BW, kg	69.8	68.1	70.3	2.1	0.74
HH, cm	89.6	90.0	90.5	0.7	0.70
HW, cm	22.9	23.2	23.2	0.3	0.74
BL, cm	83.7	84.8	85.3	1.3	0.69
Age at complete weaning	50.2	50.6	49.9	2.0	0.90
**Post-weaning**					
***First 14 d post-weaning***					
Starter feed intake, kg/d	2.21 ^a^	2.01 ^b^	1.82 ^c^	0.03	<0.01
Drinking water intake, kg/d	9.52 ^a^	9.52 ^a^	7.84 ^b^	0.16	<0.01
***At 70 d of age***					
BW, kg	91.8	88.9	89.4	1.8	0.48
HH, cm	94.3	95.1	94.3	0.7	0.70
HW, cm	24.8	24.8	24.5	0.3	0.73
BL, cm	90.1	90.0	90.5	1.1	0.95
***63 to 70 d***					
ADG, kg/d	0.77	0.92	1.08	0.07	0.08
ADG: SFI ^2^	0.29 ^b^	0.36 ^ab^	0.50 ^a^	0.07	0.02

^1^ CTL=no AA supplementation; GLN = supplementation of Gln (8.0 g/d or ~1.0% of dry matter intake); and GLNB = supplementation of Gln with BCAA (Gln = 8.0 g/d, Leu = 17.0 g/d, Ile = 10.0 g/d, and Val = 11.0 g/d). ^2^ BW = body weight; HH = hip height; HW = hip width; and BL = body length. ^a,b,c^ Least squares means with different superscripts indicate significant difference (*p* < 0.05). The bold and non-italic denote the major periods, while bold and italic denote times within major periods.

**Table 2:** Serum metabolite concentrations of dairy heifer calves undergoing a stepdown weaning scheme without AA supplementations (CTL) or with Gln (GLN) or Gln and branched-chain amino acid (GLNB) supplementations (*n* = 11).

Metabolite	Pre-Weaning ^1^			During Weaning ^2^			SEM		*p*-Value	
	CTL	GLN	GLNB	CTL	GLN	GLNB		Trt	Period	Trt × Period
Essential AA, μmol/L										
Met	0.4	0.5	0.8	0.5	0.4	0.7	0.2	0.27	0.96	0.64
Lys	12.9	17.5	9.6	17.4	11.8	10.4	3.1	0.15	0.95	0.12
Ile	28.9	29.9	36.6	39.0	37.4	44.9	3.9	0.13	<0.01	0.87
Leu	43.8	44.3	45.8	42.5	38.9	42.6	5.0	0.83	0.30	0.86
Val	78.7	86.6	88.8	83.7	81.0	94.9	8.9	0.38	0.75	0.65
Phe	2.9	4.4	4.7	5.0	4.9	5.5	1.0	0.47	0.10	0.59
Thr	22.5	27.0	23.3	24.5	24.9	22.0	2.9	0.43	0.79	0.59
Total BCAA	151.1	159.7	172.1	164.8	158.1	182.9	15.3	0.18	0.49	0.99
Gln + Glu	11.0	12.2	15.0	17.6	17.8	16.4	4.9	0.95	0.08	0.67
Gly	67.2	71.3	73.2	87.5	102.7	98.6	10.0	0.45	<0.01	0.78
Ala	35.0	41.7	38.1	38.9	42.8	29.6	5.5	0.32	0.58	0.06
Ser	30.8	29.1	29.9	33.1	33.1	28.3	3.9	0.71	0.54	0.64
Asp	0.4	0.2	0.3	0.2	0.3	0.3	0.1	0.85	0.65	0.29
Pro	30.9	34.8	31.0	16.8	28.0	18.5	5.7	0.25	0.01	0.70
Tyr	9.3	14.8	18.2	10.2	11.4	16.2	5.1	0.27	0.66	0.85
Glucose, mmol/L	5.959	6.430	6.631	5.206	5.707	5.794	0.419	0.20	<0.01	0.98
BHB, mmol/L	0.026	0.022	0.029	0.049 ^a^	0.037 ^b^	0.042 ^ab^	0.004	0.03	<0.01	0.18
Urea, mmol/L	0.556 ^b^	0.605 ^ab^	0.612 ^ab^	0.633 ^ab^	0.688 ^ab^	0.717 ^a^	0.061	0.38	0.02	0.95
Haptoglobin, µg/mL	0.75	0.58	0.63	0.95	0.81	0.82	0.19	0.52	0.07	0.98

^1^ One day before weaning began (35 d), ^2^ At the end of the first week of weaning (42 d), ^a,b^ Different superscripts in a row indicate significantly different (*p* < 0.05) means in each period separately.

## Data Availability

All datasets collected and analyzed during the current study are available from the corresponding author on fair request.

## References

[1] United States Department of Agriculture (2016). Dairy 2014, Dairy Cattle Management Practices in the United States, 2014.

[2] Urie N.J., Lombard J.E., Shivley C.B., Kopral C.A., Adams A.E., Earleywine T.J., Olson J.D., Garry F.B. (2018). Preweaned Heifer Management on US Dairy Operations: Part I. Descriptive Characteristics of Preweaned Heifer Raising Practices. J. Dairy Sci..

[3] Hawkins A., Burdine K., Amaral-Phillips D., Costa J.H.C. (2019). An Economic Analysis of the Costs Associated with Pre-Weaning Management Strategies for Dairy Heifers. Animals.

[4] Hawkins A., Burdine K.H., Amaral-Phillips D.M., Costa J.H.C. (2020). Effects of Housing System on Dairy Heifer Replacement Cost from Birth to Calving: Evaluating Costs of Confinement, Dry-Lot, and Pasture-Based Systems and Their Impact on Total Rearing Investment. Front. Vet. Sci..

[5] Campbell J.M., Crenshaw J.D., Polo J. (2013). The Biological Stress of Early Weaned Piglets. J. Anim. Sci. Biotechnol..

[6] De Passillé A.M., Borderas T.F., Rushen J. (2011). Weaning Age of Calves Fed a High Milk Allowance by Automated Feeders: Effects on Feed, Water, and Energy Intake, Behavioral Signs of Hunger, and Weight Gains. J. Dairy Sci..

[7] Jafari A., Azarfar A., Alugongo G.M., Ghorbani G.R., Mirzaei M., Fadayifar A., Omidi-Mirzaei H., Cao Z., Drackley J.K., Hossieni Ghaffari M. (2021). Milk Feeding Quantity and Feeding Frequency: Effects on Growth Performance, Rumen Fermentation and Blood Metabolites of Holstein Dairy Calves. Ital. J. Anim. Sci..

[8] Khan M.A., Weary D.M., von Keyserlingk M.A.G. (2011). Invited Review: Effects of Milk Ration on Solid Feed Intake, Weaning, and Performance in Dairy Heifers. J. Dairy Sci..

[9] Sweeney B.C., Rushen J., Weary D.M., de Passillé A.M. (2010). Duration of Weaning, Starter Intake, and Weight Gain of Dairy Calves Fed Large Amounts of Milk. J. Dairy Sci..

[10] Bovine Alliance on Management and Nutrition. https://www.aphis.usda.gov/animal_health/nahms/dairy/downloads/bamn/BAMN03_GuideFeeding.pdf.

[11] Bovine Alliance on Management and Nutrition. https://www.aphis.usda.gov/animal_health/nahms/dairy/downloads/bamn/BAMN17_GuideFeeding_1.pdf.

[12] Eckert E., Brown H.E., Leslie K.E., DeVries T.J., Steele M.A. (2015). Weaning Age Affects Growth, Feed Intake, Gastrointestinal Development, and Behavior in Holstein Calves Fed an Elevated Plane of Nutrition during the Preweaning Stage. J. Dairy Sci..

[13] Wickramasinghe H.K.J.P., Kaya C.A., Baumgard L.H., Appuhamy J.A.D.R.N. (2022). Early Step-down Weaning of Dairy Calves from a High Milk Volume with Glutamine Supplementation. J. Dairy Sci..

[14] Wu G., Meier S.A., Knabe D.A. (1996). Dietary Glutamine Supplementation Prevents Jejunal Atrophy in Weaned Pigs. J. Nutr..

[15] Xiong X., Tan B., Song M., Ji P., Kim K., Yin Y., Liu Y. (2019). Nutritional Intervention for the Intestinal Development and Health of Weaned Pigs. Front. Vet. Sci..

[16] Wu G. (2010). Functional Amino Acids in Growth, Reproduction, and Health. Adv. Nutr..

[17] Chalvon-Demersay T., Luise D., Le Floc’h N., Tesseraud S., Lambert W., Bosi P., Trevisi P., Beaumont M., Corrent E. (2021). Functional Amino Acids in Pigs and Chickens: Implication for Gut Health. Front. Vet. Sci..

[18] Habibi M., Shili C., Sutton J., Goodarzi P., Maylem E.R., Spicer L., Pezeshki A. (2021). Branched-Chain Amino Acids Partially Recover the Reduced Growth of Pigs Fed with Protein-Restricted Diets through Both Central and Peripheral Factors. Anim. Nutr..

[19] Ren M., Zhang S.H., Zeng X.F., Liu H., Qiao S.Y. (2015). Branched-Chain Amino Acids Are Beneficial to Maintain Growth Performance and Intestinal Immune-Related Function in Weaned Piglets Fed Protein Restricted Diet. Asian-Australas. J. Anim. Sci..

[20] Tokach M.D., Kats L.J., Goodband R.D., Nelssen J.L. Influence of Weaning Weight and Growth during the First Week Postweaning on Subsequent Pig Performance. Proceedings of the Swine Day, Kansas State University.

[21] Williams A.P., D’Mello J.P.F. (1994). Amino acid requirements of the veal calf and beef steer. Amino Acids in Farm Animal Nutrition.

[22] Association of Official Analytical Chemists (2000). Official Methods of Analysis of AOAC International.

[23] Van Soest P.J., Robertson J.B., Lewis B.A. (1991). Methods for dietary fiber, neutral detergent fiber, and nonstarch polysaccharides in relation to animal nutrition. J. Dairy. Sci..

[24] Ji S., Willis G.M., Scott R.R., Spurlock M.E. (1998). Partial Cloning and Expression of the Bovine Leptin Gene. Anim. Biotechnol..

[25] Melnik B.C., Schmitz G., John S.M., Carrera-Bastos P., Lindeberg S., Cordain L. (2013). Metabolic Effects of Milk Protein Intake Strongly Depend on Pre-Existing Metabolic and Exercise Status. Nutr. Metab..

[26] Duttlinger A.W., Kpodo K.R., Schinckel A.P., Richert B.T., Johnson J.S. (2020). Effects of Increasing Dietary L-Glutamine to Replace Antibiotics on Pig Health and Performance Following Weaning and Transport. Transl. Anim. Sci..

[27] Wang S., Wang F., Kong F., Cao Z., Wang W., Yang H., Wang Y., Bi Y., Li S. (2022). Effect of Supplementing Different Levels of L-Glutamine on Holstein Calves during Weaning. Antioxidants.

[28] Wickramasinghe H.K.J.P., Kramer A.J., Appuhamy J.A.D.R.N. (2019). Drinking Water Intake of Newborn Dairy Calves and Its Effects on Feed Intake, Growth Performance, Health Status, and Nutrient Digestibility. J. Dairy Sci..

[29] Johnson R.W., Patience J.F. (2012). Fueling the Immune Response: What’s the Cost?. Feed Efficiency in Swine.

[30] Lochmiller R.L., Deerenberg C. (2000). Trade-Offs in Evolutionary Immunology: Just What Is the Cost of Immunity?. Oikos.

[31] Sauerwein H., Schmitz S., Hiss S. (2005). The Acute Phase Protein Haptoglobin and Its Relation to Oxidative Status in Piglets Undergoing Weaning-Induced Stress. Redox Rep..

[32] Li J.Y., Suzuki K., Koike Y., Chen D.S., Yonezawa T., Nishihara M., Manabe N. (2005). Effects of Dietary Supplementation with Branched-Chain Amino Acids (BCAAs) during Nursing on Plasma BCAA Levels and Subsequent Growth in Cattle. Asian-Australas. J. Anim. Sci..

[33] Heng J., Wu Z., Tian M., Chen J., Song H., Chen F., Guan W., Zhang S. (2020). Excessive BCAA Regulates Fat Metabolism Partially through the Modification of M6A RNA Methylation in Weanling Piglets. Nutr. Metab..

[34] Okuro K., Fukuhara A., Minemura T., Hayakawa T., Nishitani S., Okuno Y., Otsuki M., Shimomura I. (2021). Glutamine Deficiency Induces Lipolysis in Adipocytes. Biochem. Biophys. Res. Commun..

[35] Van Thang T., Sunagawa K., Nagamine I., Kishi T., Ogura G. (2012). A Physiological Stimulating Factor of Water Intake during and after Dry Forage Feeding in Large-Type Goats. Asian-Australas. J. Anim. Sci..

[36] Burgos M.S., Langhans W., Senn M. (2000). Role of Rumen Fluid Hypertonicity in the Dehydration-Induced Hypophagia of Cows. Physiol. Behav..

[37] Krause E.G., de Kloet A.D., Flak J.N., Smeltzer M.D., Solomon M.B., Evanson N.K., Woods S.C., Sakai R.R., Herman J.P. (2011). Hydration State Controls Stress Responsiveness and Social Behavior. J. Neurosci..

[38] Calder P.C., Yaqoob P. (1999). Glutamine and the Immune System. Amino Acids.

[39] Löest C.A., Gilliam G.G., Waggoner J.W., Turner J.L. (2018). Post-Ruminal Branched-Chain Amino Acid Supplementation and Intravenous Lipopolysaccharide Infusion Alter Blood Metabolites, Rumen Fermentation, and Nitrogen Balance of Beef Steers. J. Anim. Sci..

[40] Henry Y., Sève B., Colléaux Y., Ganier P., Saligaut C., Jégo P. (1992). Interactive Effects of Dietary Levels of Tryptophan and Protein on Voluntary Feed Intake and Growth Performance in Pigs, in Relation to Plasma Free Amino Acids and Hypothalamic Serotonin. J. Anim. Sci..

[41] Tian M., Heng J., Song H., Shi K., Lin X., Chen F., Guan W., Zhang S. (2019). Dietary Branched-Chain Amino Acids Regulate Food Intake Partly through Intestinal and Hypothalamic Amino Acid Receptors in Piglets. J. Agric. Food Chem..

[42] Conigrave A.D., Quinn S.J., Brown E.M. (2000). L-Amino Acid Sensing by the Extracellular Ca2+-Sensing Receptor. Proc. Natl. Acad. Sci. USA.

[43] Ferrigno A., Berardo C., Di Pasqua L.G., Siciliano V., Richelmi P., Vairetti M. (2017). Localization and Role of Metabotropic Glutamate Receptors Subtype 5 in the Gastrointestinal Tract. World J. Gastroenterol..

[44] Liu J., Yu K., Zhu W. (2016). Amino Acid Sensing in the Gut and Its Mediation in Gut-Brain Signal Transduction. Anim. Nutr..

[45] Attele A.S., Shi Z.Q., Yuan C.-S. (2002). Leptin, Gut, and Food Intake. Biochem. Pharmacol..

[46] Hayashi H., Yamakado M., Yamaguchi M., Kozakai T. (2020). Leptin and Ghrelin Expressions in the Gastrointestinal Tracts of Calves and Cows. J. Vet. Med. Sci..

[47] Hernández-Castellano L.E., Özçelik R., Hernandez L.L., Bruckmaier R.M. (2018). Short Communication: Supplementation of Colostrum and Milk with 5-Hydroxy-l-Tryptophan Affects Immune Factors but Not Growth Performance in Newborn Calves. J. Dairy Sci..

[48] Marrero M.G., Dado-Senn B., Field S.L., da Silva D.R., Skibiel A.L., Laporta J. (2019). Increasing Serotonin Bioavailability in Preweaned Dairy Calves Impacts Hematology, Growth, and Behavior. Domest. Anim. Endocrinol..

[49] Namkung J., Kim H., Park S. (2015). Peripheral Serotonin: A New Player in Systemic Energy Homeostasis. Mol. Cells.

[50] Erspamer V. (1953). Concerning the 5-hydroxytryptamine (enteramine) content of the gastrointestinal tract lining. Naturwissenschaften.

[51] Erspamer V. (1954). Pharmacology of indole-alkylamines. Pharmacol. Rev..

[52] Twarog B.M., Page I.H. (1953). Serotonin Content of Some Mammalian Tissues and Urine and a Method for Its Determination. Am. J. Physiol..

[53] Goodarzi P., Habibi M., Roberts K., Sutton J., Shili C.N., Lin D., Pezeshki A. (2021). Dietary Tryptophan Supplementation Alters Fat and Glucose Metabolism in a Low-Birthweight Piglet Model. Nutrients.

[54] Wessels A.G., Kluge H., Hirche F., Kiowski A., Schutkowski A., Corrent E., Bartelt J., König B., Stangl G.I. (2016). High Leucine Diets Stimulate Cerebral Branched-Chain Amino Acid Degradation and Modify Serotonin and Ketone Body Concentrations in a Pig Model. PLoS ONE.

[55] Cowley M.A., Smart J.L., Rubinstein M., Cerdán M.G., Diano S., Horvath T.L., Cone R.D., Low M.J. (2001). Leptin Activates Anorexigenic POMC Neurons through a Neural Network in the Arcuate Nucleus. Nature.

[56] Sohn J.-W., Xu Y., Jones J.E., Wickman K., Williams K.W., Elmquist J.K. (2011). Serotonin 2C Receptor Activates a Distinct Population of Arcuate Pro-Opiomelanocortin Neurons via TRPC Channels. Neuron.

[57] Noaman I.M. (2015). The Effect of Serotonin on Leptin and Grelin Hormones Concentrations in Female Rats. Kufa J. Vet. Med. Sci..

